# The Protective Effects of Inulin-Type Fructans Against High-Fat/Sucrose Diet-Induced Gestational Diabetes Mice in Association With Gut Microbiota Regulation

**DOI:** 10.3389/fmicb.2022.832151

**Published:** 2022-04-14

**Authors:** Miao Miao, Qing Wang, Xinyan Wang, Chong Fan, Ting Luan, Lina Yan, Yue Zhang, Xin Zeng, Yongmei Dai, Ping Li

**Affiliations:** Nanjing Maternity and Child Health Care Hospital, Women’s Hospital of Nanjing Medical University, Nanjing, China

**Keywords:** inulin-type fructans, high-fat/sucrose diet, gut microbiota, maternal metabolism, gestational diabetes mellitus (GDM)

## Abstract

**Background:**

Inulin-type fructans (ITF) have been used as prebiotics to alleviate glucose and lipid metabolism disorders. However, few studies evaluated the microbial mechanism of ITF in improving maternal metabolic status during pregnancy.

**Methods:**

C57BL/6J mice were fed a high-fat/sucrose diet (HFD) for 4 weeks before and throughout pregnancy to induce a model of gestational diabetes mellitus (GDM). Body weight, glycolipid metabolic parameters, and fecal short-chain fatty acids (SCFAs) were assessed in the experimental process. The effects of ITF on the fecal microbiota were analyzed by 16S rRNA gene amplicon sequencing.

**Results:**

Pregnant HFD-fed mice displayed significant insulin resistance and dyslipidemia. ITF (3.33 g/kg/day) treatment improved glucose and lipid metabolism disorder parameters in HFD-induced GDM mice and alleviated fat accumulation and glucose intolerance. The alpha diversity of the gut microbial community was increased in ITF mice, while the beta diversity returned to the level of normal chow diet (NCD) mice. Interestingly, Verrucomicrobia, *Bifidobacterium*, and *Akkermansia* were obviously enriched, while *Dubosiella* was obviously lessened after inulin treatment. Further analysis indicated that *Dubosiella* was positively correlated with markers of glycolipid metabolism disorders, whereas the ITF-supplemented diet partially reversed the changes.

**Conclusion:**

Our results suggest that the ITF treatment may alleviate glucose and lipid metabolism disorders with the mediation of gut microbiota.

## Background

Gestational diabetes mellitus (GDM), carbohydrate intolerance, and insulin resistance during pregnancy are serious problems with increasing prevalence (American Diabetes, 2019), resulting in significant short-term and long-term adverse health outcomes in both mother and offspring (Miao et al., 2017; Song et al., 2018; Lowe, 2019). The physiological changes in insulin resistance and lipid profiles are exacerbated in women with GDM and may indicate an underlying metabolic dysfunction that transiently manifests during pregnancy (Schneider et al., 2011; Zhu and Zhang, 2016).

Gut dysbiosis plays a vital role in abnormal host metabolism, as recently demonstrated in studies of type 2 diabetes (T2D) and obesity (Karlsson et al., 2013). *Prevotella* and *Bacteroides* have been identified as the main species contributing to insulin resistance and glucose intolerance (Pedersen et al., 2016). While the impact of gut microbiota on host metabolism and metabolic diseases is well-documented (Moller, 2001), only recently have studies focused on microbiota changes that influence metabolic mechanisms during pregnancy (Koren et al., 2012). *Parabacteroides* are significantly more abundant in GDM women than in healthy pregnant women (Kuang et al., 2017). A novel relationship between gut microbiome composition and the metabolic hormonal environment in overweight and obese pregnant women at the first trimester has also been described (Gomez-Arango et al., 2016). These studies suggest that major shifts in the gut microbiome during pregnancy may play a crucial part in the development of GDM.

Dietary intervention has become a potentially effective strategy to modulate the gut microbiota and improve the host health (Marchesi et al., 2016). Inulin-type fructans (ITF) are a type of dietary fiber present in vegetables, such as chicory roots, and can also be extracted to be used as food ingredients (Kalala et al., 2018). Isolated ITF have been considered to be typical prebiotics (Gibson et al., 2017). Prebiotics are defined as non-digestible compounds that are generated through fermentation by the gut. Prebiotics are able to modulate the composition and/or activity of the gut microbiota, thereby conferring a beneficial physiological effect on the host (Bindels et al., 2015; Salminen et al., 2021). *In vitro* studies and randomized controlled trials have shown that ITF can stimulate the growth of *Bifidobacterium* populations (Roberfroid et al., 1998; Sawicki et al., 2017) and certain butyrate-producing species (Ramirez-Farias et al., 2009; Scott et al., 2014) as well as reduce the abundance of Firmicutes (Everard et al., 2011, 2013; Dewulf et al., 2013). In addition, numerous randomized controlled trials have demonstrated direct health benefits of ITF, including inhibiting pathogens, protecting against cardiovascular diseases, and improving mineral bioavailability (Abrams et al., 2005; Kellow et al., 2014; Lohner et al., 2014). However, the relationships among dietary ITF, GDM and gut microbiota are still not clear.

Given that there are few studies aiming to evaluate the microbial mechanism of soluble dietary fiber in improving maternal metabolic status during pregnancy, our current research was undertaken to investigate the effects of adding ITF to a high-fat/sucrose diet (HFD) on the composition and metabolites of fecal microbiota from 4 weeks before conception and throughout gestation as well as maternal and neonatal health parameters in a GDM mouse model. In human intervention studies, doses ranging from 12 to 16 g/day are often given when testing for metabolic effects of ITF (Cani et al., 2009; Parnell and Reimer, 2009; Tarini and Wolever, 2010; Rahat-Rozenbloom et al., 2017), which equals to 2.46 and 3.33 g/kg body weight in mice, respectively (Nair and Jacob, 2016). In this study, the dose was based on our previously published study of ITF administration in mice (Miao et al., 2021). A high dose was chosen due to the short window of treatment allowed by pregnancy. Our study aimed to provide some microbial mechanistic insights into the application of ITF to a typical gestational diet characterized by high-fat/sucrose for improving maternal and neonatal health.

## Materials and Methods

### Materials

Six-week-old C57BL/6J mice were purchased from Vital River Laboratory Animal Technology Co., Ltd. (Beijing, China). ITF were procured from Fengning Ping’an hi tech Industry Co., Ltd. (Hebei, China), Thermo Scientific (Massachusetts, United States), Thermo Fisher (Massachusetts, United States), (Vilof™ soluble dietary fiber powder) which contains 91% ITF and 9% mixture of sucrose, fructose, and glucose.

### Animal Treatment and Experiment Design

Mice were housed in a temperature- and humidity-controlled laboratory. This animal experiment was approved by the Animal Protection Ethics Committee of Women’s Hospital of Nanjing Medical University (No. 2018-49). All animal experiments were performed in accordance with Chinese national regulations on the administration of animal experimentation as well as international guidelines on animal experimentation. After 1 week of acclimatization, mice were randomly divided into three groups (*n* = 5): control [normal chow diet (NCD) + vehicle, *n* = 5], HFD (HFD + vehicle, *n* = 5), and ITF treatment (HFD + ITF, *n* = 5). In order to compare the changes of fecal microbiota before and after pregnancy, the three groups were renamed to normal chow diet in gestation (NCDG) group, HFDG group, and ITFG group after mating. The NCD mice were fed a low-fat diet (Research Diet AIN-93G, consisting of 20.3% protein, 63.9% carbohydrate, and 15.8% fat) for 4 weeks prior to mating and throughout pregnancy (18 days), while both HFD and ITF treatment groups were fed an HFD (Research Diet D12451, consisting of 35.2% protein, 63.9% carbohydrate, and 45% fat). The ITF treatment group received a dose of 3.33 g/kg of ITF each day *via* oral gavage, while the NCD and HFD groups received the same dose of a vehicle (DD H_2_O). All mice were given free access to 100 g of fresh diet and 250 ml of fresh water daily per cage (five mice per cage).

### Fasting Blood Glucose and Oral Glucose Tolerance Test

Blood samples were collected from the tail vein, and blood glucose levels were measured with a glucose meter (Roche Accu-Chek Active, Mannheim, Germany). FBG was monitored at different time points, including before dietary intervention, after 4 weeks of HFD, and on gestational age of 0 day (GD0), gestational age of 10 days (GD10), gestational age of 14 days (GD14), and gestational age of 18 days (GD18). OGTT was performed on GD14. The animals fasted for 6 h and then were gavaged with 2 g/kg glucose. The blood glucose levels at 0, 30, 60, 90, and 120 min were determined.

### Detection of Biochemical Indexes

Mice were euthanized by CO_2_ inhalation on GD18 (or equivalent) after fasting for 6 h from 8 a.m., and blood sample was collected. Blood was centrifuged at 3,000 *g* for 15 min at 4°C, and serum was isolated. The levels of fasting serum insulin (FINS), triglyceride (TG), total cholesterol (TC), low-density lipoprotein (LDL), and high-density lipoprotein (HDL) were measured using a commercial detection kit (NJJCBIO Co., Ltd., Nanjing, China) according to the kit instructions.

Based on the measured content of FBG and FINS, the homeostasis model of assessment (HOMA) for insulin resistance (IR) index (HOMA-IRI) was calculated and compared. HOMA-IRI was calculated as [fasting glucose (mmol/L) × fasting insulin (mU/L)]/22.5. Meanwhile, the area under the curve (AUC) of blood glucose was calculated (Lachine et al., 2016).

### Hematoxylin–Eosin Staining

Liver and inguinal fat tissues were fixed in 4% paraformaldehyde, decalcified, paraffin embedded, and stored at 4°C. After tissues were sliced into 4 μm sections, hematoxylin–eosin staining was performed. First, sections were stained with hematoxylin for 5–10 min, immersed in 70% ethanol for 30 min to remove cytoplasm coloring, alkalized with alkaline solution, and washed with distilled water for 1 min. Second, sections were stained with eosin for 30–60 s, dehydrated with gradient ethanol, cleared two times with xylene, dried, and mounted. Finally, the morphological structures of the liver and inguinal fat tissues were observed under an optical microscope.

### Fecal DNA Extraction

One day prior to mating and GD18, fecal samples were collected in individual sterilized cages and immediately frozen in liquid nitrogen. About 100 mg of stool samples was used to extract total genome DNA according to the DNA extraction kit (DP328, Tiangen Company, Beijing, China). The concentration and purity of the extracted bacterial DNA were detected using a Qubit 2.0 fluorometer (Thermo Scientific, United States). The 16S rRNA gene V4 region-specific primers are 515F (GTGCCAGCMGCCGCGGTAA) and 806R GGACTACHVGGGTWTCTAAT. The PCR products of sterile water were considered as the negative control for 16S rRNA sequencing. The PCR products were purified using the Gene JET Gel Extraction Kit (Thermo Scientific). The library was constructed using Ion Plus Fragment Library Kit 48 reactions (Thermo Fisher, United States). After Qubit quantification and testing, the library was sequenced by Thermo Fisher’s Ion S5™ XL.

### Gut Microbiota Analysis

Raw data were obtained after data processed using Cutadapt (V1.9.1^1^). Then, chimera sequences were removed to obtain clean reads. Operational taxonomic units (OTUs) were assigned for sequences with ≥97% similarity. OTUs were annotated using the SILVA132 database.^2^ The taxonomic information was obtained, and the community composition was counted at seven taxonomic levels: kingdom, phylum, class, order, family, genus, and species. Alpha diversity was analyzed by Chao 1^3^ with QIIME software (version 1.9.1). Beta-diversity metrics were calculated by the non-metric multidimensional scaling (NMDS) model based on the Bray--Curtis distance. One-way analysis of similarities (ANOSIM) with multiple pairwise post-tests on all groups at the same time was performed to test whether the difference between the extra groups was greater than that between the intra-groups and to assess the significance of the difference in separation. Chao 1, Bray--Curtiss indexes, NMDS, and ANOSIM were calculated at the OTU level. Differentially abundant genera were analyzed by meta stats^4^ with a non-parametric test, followed by the Benjamini and Hochberg false discovery rate approach to filter relevant *p*-values.

### Fecal Short-Chain Fatty Acid Analysis

The feces from each mouse were collected and frozen at −80°C. Acetate, propionate, and butyrate in fecal samples were analyzed using gas chromatography–mass spectrometry (GC-MS) (Sun et al., 2015). Briefly, the feces were homogenized with a saturated sodium chloride solution and acidified with 10% sulfuric acid. Next, diethyl ether was used to extract SCFAs. After centrifugation, the supernatants were harvested for GC-MS.

### Statistical Analysis

Data represent mean ± standard error of the mean. For parametric variables, the unpaired two-tailed Student *t*-test was used to assess the differences in mean values between two groups. For three groups, statistical analysis was performed with ANOVA with Tukey *post hoc* test. For non-parametric variables, the statistical significance of the differences was evaluated by the Mann–Whitney test or Kruskal–Wallis test. For the OGTT, two-way ANOVA was performed for the evolution of blood glucose levels with a *post hoc* test using the Bonferroni method. A *p*-value < 0.05 was considered statistically significant. GraphPad Prism 7 (GraphPad Software, San Diego, CA, United States) was used to do the statistical analyses.

## Results

### Changes of Body Weight and Glycolipid Metabolic Parameters in Mice

**FIGURE 1:**
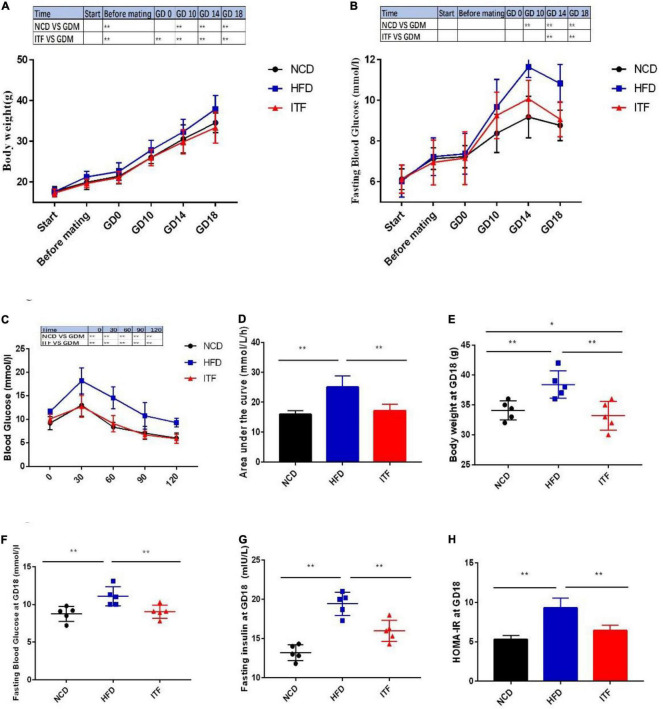
Improvement in metabolic parameters in HFD-induced gestational diabetes mice by ITF. **(A)** Body weight. **(B)** FBG. **(C)** Plasma glucose profile. **(D)** Mean AUC measured during the OGTT. **(E)** Body weight at GD18. **(F)** FBG at GD18. **(G)** Fasting insulin at GD18. **(H)** HOMA-IR at GD18. AUC, area under the curve; OGTT, oral glucose tolerance test. Data are presented as mean ± SEM. Data were analyzed using two-way ANOVA followed by the Bonferroni *post hoc* test for panels **(A,B,F)** and using one-way ANOVA followed by the Tukey *post hoc* test for panels **(C–E,G)**. *p < 0.05, **p < 0.01.

**FIGURE 2:**
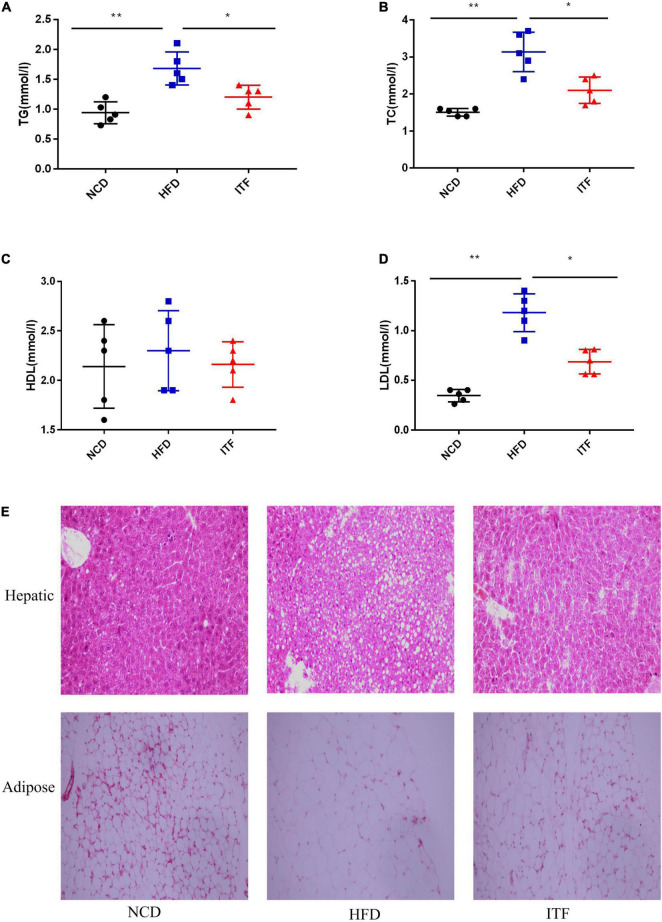
Improvement in metabolic parameters in HFD-induced gestational diabetes mice by ITF. **(A)** Serum TG. **(B)** Serum TC. **(C)** Serum HDL-C. **(D)** Serum TC LDL-C. **(E)** Representative H&E-stained images of the hepatic and adipose tissues (×200). TG, triacylglycerol; TC, total cholesterol; HDL-C, high-density lipoprotein cholesterol; LDL-C, low-density lipoprotein cholesterol. Data were analyzed using one-way ANOVA followed by the Tukey *post hoc* test for panels **(A–D)**. *p < 0.05, **p < 0.01.

To investigate the effect of inulin treatment on glycolipid metabolism disorders in HFD-induced gestational diabetes mice, we examined the body weight, daily food intake, and glycolipid metabolism-related parameters. The body weight, FBG, FINS, HOMA-IR, TG, TC, LDL-C, and the AUC of OGTT of the HFDG group mice were significantly elevated compared with those of the NCDG group mice (Figures 1A–H, 2A–D), indicating severe glucose intolerance, insulin resistance and dyslipidemia.

In contrast, ITFG group mice fed the ITF-supplemented diet showed improved metabolic parameters (Figures 1A–G, 2A–D). After ITF intervention, body weight, serum TG, TC, and LDL-C on GD18 reduced significantly by 4.54 g, 0.48 mmol/l, 1.04 mmol/l, and 0.494 mmol/l (*p* < 0.05, vs. HFDG group) (Figures 1C, 2A,B,D), respectively. Additionally, the AUC of OGTT on GD14 and the FBG and serum insulin on GD18 were lowered by 7.95 mmol/L/h, 2.04 mmol/l, and 3.46 mIU/L, respectively (*p* < 0.05, vs. HFDG group), indicating a significant improvement in glucose tolerance (Figures 1D,E,G). According to hepatic and adipose tissue staining (Figure 2E), the HFDG group mice exhibited severe hepatic lipid droplets and adipocyte hypertrophy, which were alleviated after ITF treatment. Overall, the above results indicate that ITF have a beneficial effect that ameliorates glycolipid metabolism disorders in HFD mice.

### Reproductive Outcomes of Pregnant Mice

The number, body weight, and length of fetal mice in each group were compared. There was no significant difference in the number of fetal mice among groups (Supplementary Figure 1A). The average body weight and length of fetal mice born by HFD mothers (1.26 ± 0.16 g and 2.33 ± 0.09 cm, respectively) were significantly higher than those by ITF mothers (1.03 ± 0.05 g and 2.16 ± 0.07 cm, respectively) (Supplementary Figures 1B,C).

### Changes of Fecal Microbial Diversity

**FIGURE 3:**
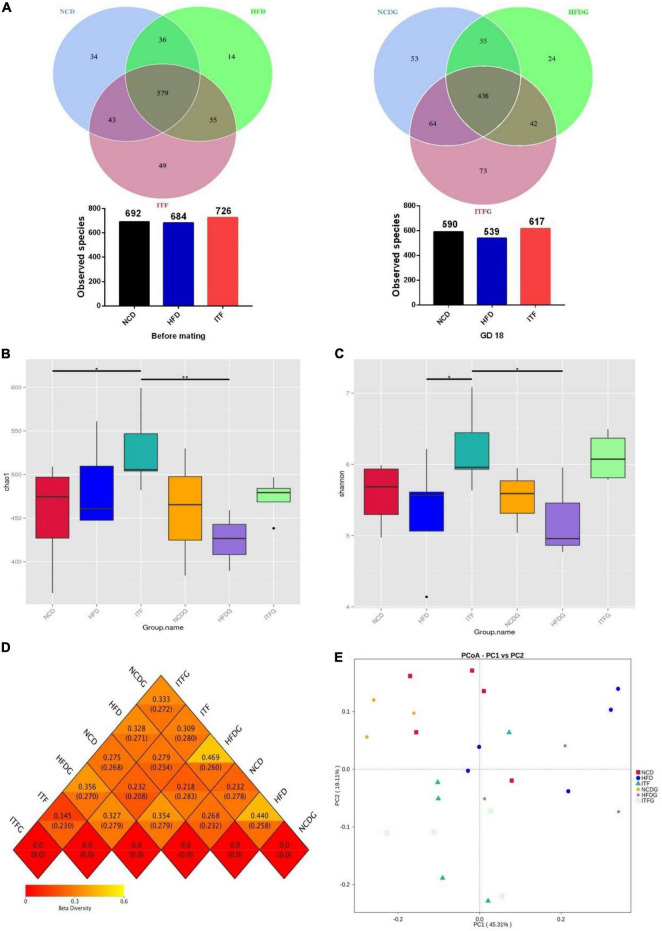
ITF modify the composition of the cecal microbiota in ob/ob mice. **(A)** OTU number before mating and on GD18. **(B)** Chao 1 index of microbiota. **(C)** Shannon index of microbiota. **(D)** Heat map of beta diversity index. **(E)** The beta diversity of gut microbiota analyzed by PCoA. Data were analyzed using one-way ANOVA followed by the Tukey *post hoc* test for panels **(B,C)**. *p < 0.05, **p < 0.01.

We used the 16S rRNA gene amplicon sequencing method (V4 region) and generated 2,131,728 reads for a total of 25 samples, with an average of 85,269 ± 22,171 reads per sample. At each stage, NCD-HFD-ITF and NCDG-HFDG-ITFG pairs shared less common OTUs with each other. The Venn graph exhibited common OTUs for NCD-HFD-ITF and NCDG-HFDG-ITFG pairs, decreasing from 579 before mating to 438 on GD18 (Figure 3A).

To assess the fecal microbial community structure, richness (Chao 1 index) and diversity (Simpson index) were calculated (Figures 3B,C). For Chao 1 index, the data of the ITF group were significantly higher than those of NCD and HFDG groups (*p* < 0.05, *p* < 0.01). A remarkable increment in Simpson index with ITF supplementation was found compared with HFD and HFDG groups in the present study (*p* < 0.05). All the results above provided the view that ITF treatment could effectively improve the decline of Chao 1 index and Simpson index induced by HFD addition.

We then used principal co-ordinate analysis (PCoA) to categorize the OTU data into two main factors that explained 64.42% of the variance (Figure 3E), which showed that the microbiomes in NCD (NCD and NCDG), HFD (HFD and HFDG), and ITF (ITF and ITFG) treatment groups significantly differed from one another while the two groups of the same treatment shared some overlapping regions before and after conception, which indicated that the overall gut microbial community had been significantly modified. The four groups exhibited significant, tight clustering according to NCD or ITF diet. Independent biological replicates were generally consistent, but more variable among mice fed by HFD (Figure 3D).

### Changes of the Relative Abundance at the Phylum Level

**FIGURE 4:**
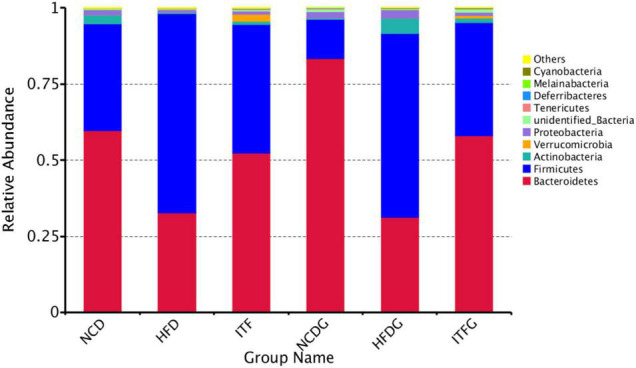
Relative abundance of microbial species of the top 10 phyla in the feces of mice.

The phylum Bacteroidetes was dominant among the nine phyla (>1% in at least one sample) present in the gut microbiota from the six groups of mice, and the ratio of Firmicutes/Bacteroidetes (F/B) was increased in HFD and HFDG mice over the NCD and NCDG groups, but lower in the ITF and ITFG groups compared with HFD and HFDG mice (Figure 4 and Supplementary Table 1). The gut microbiota in obese individuals has usually shown an increased F/B ratio (Ramirez-Farias et al., 2009). Therefore, the decreased F/B ratios of ITF and ITFG mean that the feature in HFD mice could be reversed by the ITF-supplemented diet. HFD treatment decreased the relative abundance of Proteobacteria before mating (*p* < 0.01). ITF supplementation increased the relative abundance of Verrucomicrobia compared with HFD before mating and on GD18 (*p* < 0.01). Relative abundances of the Deferribacteres group of HFD and the Cyanobacteria group of NCD were not detected in fecal samples on GD18. Moreover, relative abundances of Actinobacteria decreased in HFD before mating but increased substantially when reaching the perinatal period. The majority of genera were affected by the gestation stage, indicating that their relative abundances changed greatly over the pregnancy progress.

### Changes of the Relative Abundance at the Genus Level

**FIGURE 5:**
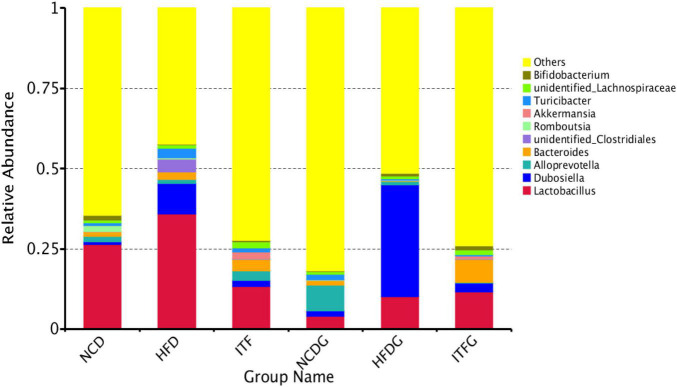
Relative abundance of microbial species of the top 10 genera in the feces of mice.

**FIGURE 6:**
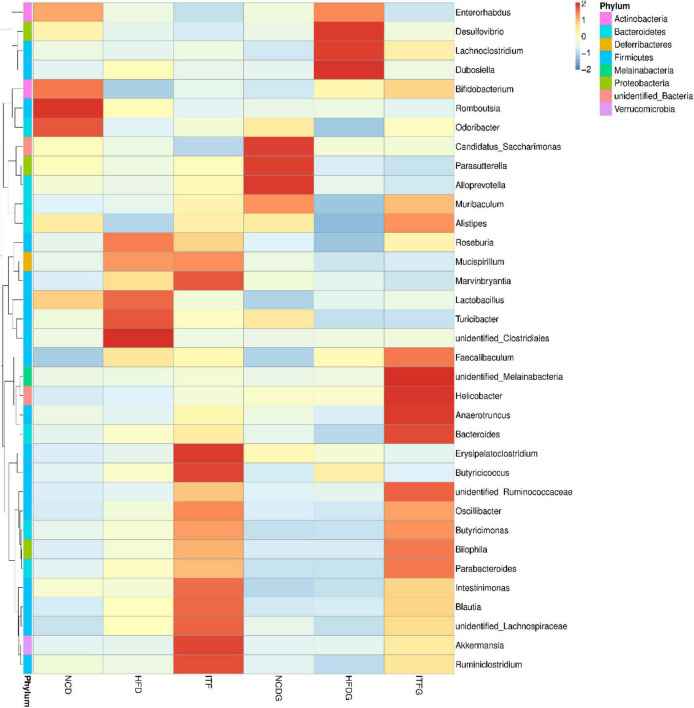
Heat map of microbial species of at the genus level in the feces of mice.

The relative abundances at the genus level (>1% in at least one sample) were present in Figure 5 and Supplementary Table 2. Fat addition (HFD and HFDG) increased the relative abundances of *Dubosiella* and *Lactobacillus* and decreased those of *Romboutsia* and *Alloprevotella* compared to the NCD (NCD and NCDG). The abundance of *Bifidobacterium* increased, whereas that of *Dubosiella* decreased with the intervention of ITF before and after conception. Our results also indicated that the abundance of *Akkermansia* was significantly higher in the ITF-treated (ITF and ITFG) groups than in any other group. The heat map analysis of microbial community composition at the family level confirmed that the abundance of *Dubosiella* that causes obesity and metabolic syndrome-related inflammation was reduced after ITF treatment (Figure 6).

**FIGURE 7:**
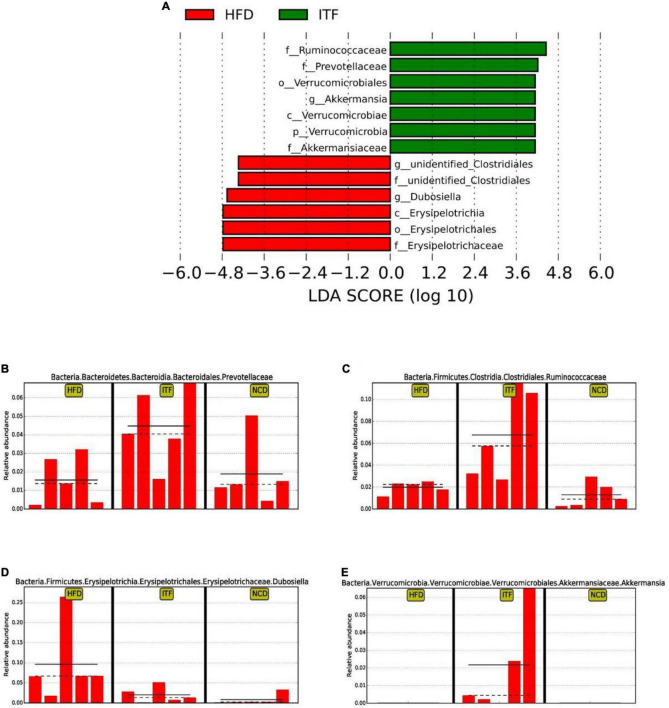
Identification of the most differentially abundant among HFD, ITF, and NCD analyzed by the LEfSe method. **(A)** LDA scores of differentially abundant taxa. **(B)** Relative abundance of Prevotellaceae. **(C)** Relative abundance of Ruminococcaceae. **(D)** Relative abundance of *Dubosiella*. **(E)** Relative abundance of *Akkermansia*.

**FIGURE 8:**
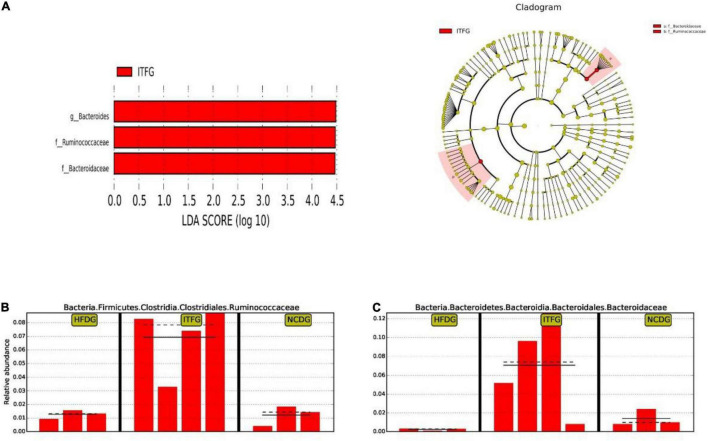
Identification of the most differentially abundant among HFDG, ITFG, and NCDG analyzed by the LEfSe method. **(A)** LDA scores of differentially abundant taxa. **(B)** Relative abundance of Ruminococcaceae. **(C)** Relative abundance of Bacteroidaceae.

Next, to identify the changes in specific bacterial taxa after ITF-supplemented diet intervention before and after conception, we utilized the linear discriminant analysis (LDA) effect size (LEfSe) to compare the fecal microbiota composition between the NCD, HFD, and ITF groups; the LDA score was selected to discriminate specific taxa in different groups. Compared with the HFD group, the ITF mice had a higher abundance of f-Ruminococcaceae, f-Prevotellaceae, o-Verrucomicrobiales, *g-Akkermansia*, c-Verrucomicrobiae, p-Verrucomicrobia, and f-Akkermansiaceae but lower abundance of *g-Unidentified clostridiales*, *f-Unidentified clostridiales*, *g-Dubosiella*, c-Erysipelotrichia, o-Erysipelotrichales, and f-Erysipelotrichaceae (Figures 7A–E). Correspondingly, *g-Bacteroides*, f-Ruminococcaceae, and f-Bacteroidaceae were enriched in the ITFG group on GD18 (Figures 8A–C).

### Changes in Fecal Short Chain Fat Acids Levels Upon Inulin-Type Fructans Intervention

**FIGURE 9:**
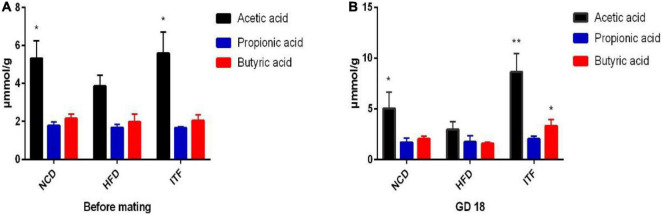
**(A)** SCFA data before mating. **(B)** SCFA data on GD18. Data were analyzed using one-way ANOVA followed by the Tukey *post hoc* test for panels **(A,B)**. *p < 0.05, **p < 0.01 compared to HFD.

Acetate, propionate, and butyrate levels in fecal samples were quantified by GC-MS. Over time, fecal acetic acid levels were significantly increased in ITF group mice when compared to HFD group mice before mating (*p* < 0.05) and on GD18 (*p* < 0.01) (Figures 9A,B and Supplementary Table 3). Butyric acid levels were significantly increased in ITF group mice compared to HFD group mice on GD18 (*p* < 0.05) (Figure 9B and Supplementary Table 3). However, we observed no differences in the propionate levels among the three groups at any of the time points (Figures 9A,B and Supplementary Table 3).

### Correlations Between Glycolipid Metabolism Indicator and Bacterial Abundance

**FIGURE 10:**
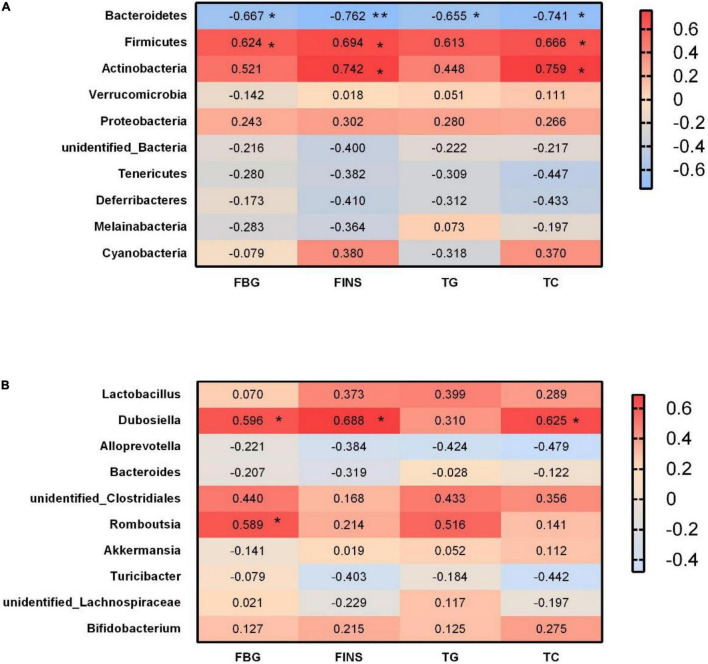
Correlations between glycolipid metabolism indicator and bacterial abundance. **(A)** Heat map of Spearman correlations between the levels of metabolites/components and the abundances of gut microbial phyla. **(B)** Heat map of Spearman correlations between the levels of metabolites/components and the abundances of gut microbial genera. FBG, fasting blood glucose; FINS, fasting insulin; TG, triglyceride; TC, total cholesterol. *p < 0.05, **p < 0.01.

At the phylum level, we analyzed the correlations between significant glycolipid metabolism indicator and gut microbiota on GD18. Bacteroidetes abundance was negatively correlated with FBG, FINS, TG, and TC, whereas Firmicutes abundance was positively correlated with FBG, FINS, and TG (Figure 10A). Moreover, Actinobacteria abundance was positively correlated with FINS and TC (Figure 10A).

At the genus level, the relative abundance of *Dubosiella* was positively correlated with FBG, FINS, and TC (Figure 10B). *Romboutsia* abundance was positively correlated with FBG (Figure 10B).

## Discussion

Gut microbiota disorder has been considered as one of the contributing factors for metabolic disorders. The composition of the microbiome also changes during pregnancy. It has been recently proposed that fecal microflora and their metabolic activities may play a critical role in body weight control, energy homeostasis, fermentation, and absorption of non-digestible carbohydrate, as well as in the development of IR. Therefore, gut microbiota may also participate in the pathogenesis of several metabolic disorders, such as obesity, diabetes mellitus, and GDM (Cani et al., 2014; Zhang et al., 2015; Rowland et al., 2018; Cortez et al., 2019). Prebiotics can exert positive effects on the maintenance of host metabolic homeostasis, which are mainly mediated by the gut microbiota (Khanum et al., 2000; Wang et al., 2021). ITF, one of the crucial prebiotics, have been demonstrated to be effective in the treatment of T2DM (Dehghan et al., 2014a; Zhang et al., 2018), while data on the effects of symbiotic supplementation on markers of insulin metabolism and lipid concentrations in GDM are scarce. The aim of this study was to determine whether ITF taken before and during pregnancy could impact the development of HFD-induced glucose intolerance during pregnancy.

To induce features of GDM, C57BL/6J mice were fed an HFD for 4 weeks before and during pregnancy. This model has previously been used to induce features of GDM in mice, such as insulin resistance and dyslipidemia (Holemans et al., 2004; Jones et al., 2009; Liang et al., 2010). A period of only 4 weeks of HFD exposure before pregnancy is not sufficient to cause a diabetic phenotype; however, continued feeding throughout pregnancy leads to progressive glucose intolerance and insulin resistance, mimicking human disease. This mouse model allowed a factorial design to determine the interaction of treatments, as well as more thorough examination of potential mechanisms and whole-tissue analysis, which would not be possible in human trials.

In the present study, we chose the dose of 3.33 g/kg/day of ITF, which was equal to the highest dose reported for human consumption (16 g/day) to evaluate the potential antidiabetic effects of ITF in GDM mice. Consistent with a previous study showing that ITF administration significantly lowered the levels of FBG, IL-6, TNF-α, and plasma LPS in T2DM patients (Dehghan et al., 2014b), we found that ITF relieved the gestational diabetic symptoms as evidenced by reduced body weight, blood glucose level, and insulin level. However, Farhangi et al. (2016) found that chicory inulin significantly reduced the fasting serum glucose level and HbA1C ratio but had little effect on the insulin level in patients with T2DM. We speculate that the different effects of chicory inulin on insulin may be due to different dosages (10 g/day for T2DM patients in Farhangi et al.’s study). Moreover, a strong hypolipidemic effect of ITF in GDM mice was observed. These results agree with a previous study showing that inulin promoted lipid metabolism by altering the expression of acetyl-CoA carboxylase and the activities of fatty acid synthase and xanthine oxidase (Lin et al., 2014).

Accumulating studies have been performed to reveal the underlying mechanisms of efficient treatment of ITF in GDM. The majority of mechanisms are attributed to gut microbiota alteration, immune inflammation, abnormal lipid metabolism, and oxidative stress. Growing evidences have demonstrated that the gut microbiota play a critical role in the development of GDM (Fugmann et al., 2015; Mokkala et al., 2017; Crusell et al., 2018; Hasan and Aho, 2018). In the present study, the alpha diversity index that was reduced by HFD could be effectively improved by inulin treatment. Stanislawski et al. (2017) reported that gestational weight gain was associated with lower alpha diversity. Beta-diversity analysis of unweighted UniFrac illustrated the distinct clustering of the relative abundances of OTUs after ITF treatment. Similar results were obtained from PCoA.

At the phylum level, a higher ratio of F/B was observed in the HFD group, which was supported by a study showing that the F/B ratio in overweight human adults was lower than that in lean controls (Lordan and Thapa, 2020). An imbalance in the F/B ratio is related to dysbiosis conditions (Ley et al., 2005; Nelson et al., 2010). The decreased F/B ratio of ITF and ITFG means that this feature in obesity could be reversed by the ITF-supplemented diet. Our analyses showed, after ITF treatment, an enhancement of the relative abundance of Verrucomicrobia in the HFD group before mating and on GD18, as well as an obviously lessened Actinobacteria on GD18. Verrucomicrobia is a member of the PVC (Planctomycetes–Verrucomicrobia–Chlamydiae) superphylum, which includes phylogenetically related bacteria with unusual characteristics such as the existence of a complex and dynamic endomembrane system that, in some aspects, makes them closer to eukaryotic cells. A recent study showed that the healthy Chilean subjects reveals a high abundance of the phylum Verrucomicrobia (Fujio-Vejar et al., 2017). Positive correlations of Actinobacteria with FINS caused aggravation of insulin resistance in the disease, which was reversed by inulin intervention.

At the genus level, ITF supplementation showed a significant effect on increasing the abundance of *Bacteroides*, which have been demonstrated to ameliorate inflammation in recent studies (Ejtahed et al., 2016; Li et al., 2017; Biruete et al., 2021). SCFAs, including acetate, propionate, and butyrate, derived from the gut microbiome are pivotal for rectifying host metabolism and immunity (Meijer et al., 2010). In the present study, we observed that acetic acid levels of the ITF group increased significantly before mating and on GD18, whereas butyric acid levels only increased on GD18, suggesting that changes of bacterial metabolites might be dependent on the intervention time. Consistent with our findings, ITF-fed mice increased the production of SCFAs, benefiting the balance of gut microbiota in the alleviation of diabetic mice (Chen et al., 2017). Significant elevation of SCFA-generating *Bacteroides* revealed that our ITF treatment may restore gut dysbiosis by promotion of *Bacteroides*. Another genus that we found increased abundance in ITF-fed mice was *Akkermansia*. Recent studies described this as an important probiotic genus, with systemic beneficial effects to the host (Cani, 2014; Cani and de Vos, 2017), including the control of metabolic syndromes (Christiansen, 2013; Dao et al., 2016). In rodents, probiotics supplementation with *Akkermansia* improved glucose tolerance and insulin sensitivity (Zhao et al., 2017). Our results suggest that *Akkermansia* might have another impact on host physiology during pregnancy than otherwise described or that we found another subspecies of *Akkermansia*. The applied 16S rRNA gene amplicon sequencing methods does, however, not make it possible to investigate this finding at a deeper taxonomic resolution. We observed that HFD mice have increased *Dubosiella*, which has been previously described in dysbiosis conditions such as GDM and obesity (Bai et al., 2019; Li et al., 2020; Sheng et al., 2020; Qiu and Macchietto, 2021; Yi et al., 2021). Positive correlations of *Dubosiella* with FBG, FINS, and TC demonstrated that these bacteria may promote the glycolipid metabolism disorders, which could be reversed by ITF treatment.

Modulation of the human gut microbiome with dietary interventions has been extensively studied, mainly focusing on the supplementation of non-digestible carbohydrates (NDCs) (Ladirat et al., 2014; Elison et al., 2016; Zhao and Zhang, 2018). However, the impact of dietary components on the stability and resilience of the gut ecosystem has been barely addressed. We found ITF intervention evidently primed the mice with significant change in microbiota profile, and the gestational impact (IFTG-IFT) was largely ameliorated compared to the other two treatments. This may be partially due to the ability of ITF to improve gut microbiome resilience. A high microbial diversity, as well as the increase of the levels of fecal SCFA, seemed to be critical aspects for the resilience of ITF group mice. Thus, further studies are required to reveal the precise mechanism(s) behind these effects.

In summary, we show that ITF treatment (3.33 g/kg/day) alleviates glucose and lipid metabolism disorders in HFD-induced gestational diabetes mice. These actions are likely to be mediated *via* increasing the abundance of Verrucomicrobia, *Bifidobacterium*, and *Akkermansia* and reducing the abundance of *Dubosiella*. We further demonstrate that the abilities of inulin intervention to enhance the relative abundance of SCFA-producing bacteria and increase the levels of SCFAs play a key role in antidiabetic effects. Our findings suggest a potential value of ITF as an inexpensive supplement for the prevention and treatment of GDM patients.

## Data Availability Statement

The original contributions presented in the study are publicly available. This data can be found here: The sequences are available in the NCBI database (Accession number: Bioproject PRJNA789154; SRA submission: SUB10794397).

## Ethics Statement

The animal study was reviewed and approved by Animal Protection Ethics Committee of Women’s Hospital of Nanjing Medical University (No. 2018-49). Written informed consent was obtained from the owners for the participation of their animals in this study.

## Author Contributions

MM and XZ conceived and designed the experiments. XW, CF, and TL performed the animal experiments and completed the analysis of metabolic parameters. QW, YZ, and LY collected the data and performed the bioinformatics and statistical analysis. MM, XW, and QW wrote the initial manuscript. XZ, YD, and PL reviewed and edited the manuscript. All authors read and approved the final manuscript.

## Conflict of Interest

The authors declare that the research was conducted in the absence of any commercial or financial relationships that could be construed as a potential conflict of interest.

## Publisher’s Note

All claims expressed in this article are solely those of the authors and do not necessarily represent those of their affiliated organizations, or those of the publisher, the editors and the reviewers. Any product that may be evaluated in this article, or claim that may be made by its manufacturer, is not guaranteed or endorsed by the publisher.

## Footnotes

1
http://cutadapt.readthedocs.io/en/stable/

2
http://www.arb-silva.de/

3
http://scikit-bio.org/docs/latest/generated/skbio.diversity.alpha.chao1.html#skbio.diversity.alpha.chao1

4
http://metastats.cbcb.umd.edu/detection.html
