# Supplementary material for: The Protective Effects of Inulin-Type Fructans Against High-Fat/Sucrose Diet-Induced Gestational Diabetes Mice in Association With Gut Microbiota Regulation
Source: Front Microbiol. 2022 Apr 14;13:832151. doi: 10.3389/fmicb.2022.832151 (PMC9048744; doi:10.3389/fmicb.2022.832151)
Supplement: Supplementary file 1 [file Data_Sheet_1.PDF]

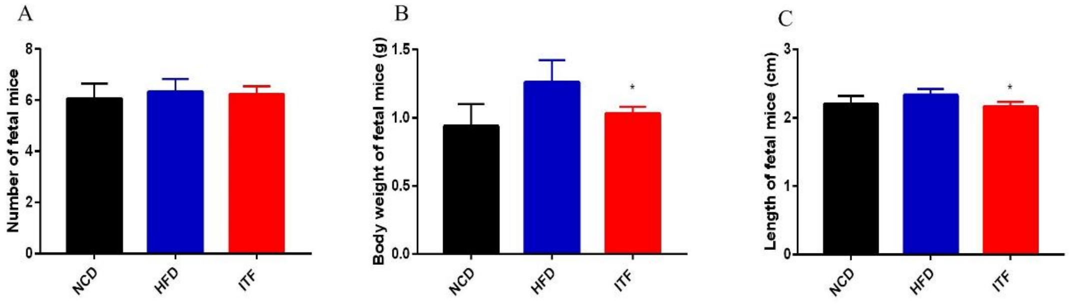

Figure S1 Reproductive outcomes of pregnant mice

Changes of the number(A), length of fetal mice (B) and body weight at birth (C). Data are expressed as the mean ± SD values (n = 15). **P* <0.05 vs HFD group.

Table S1 Relative abundance of microbial species of the top 10 phylum in the feces of mice

	NCD	HFD	ITF	NCDG	HFDG	ITFG
Bacteroidetes	0.596879	0.326761	0.523311	0.833583	0.311923	0.580551
Firmicutes	0.350518	0.655052	0.423544	0.128789	0.603914	0.372607
Actinobacteria	0.030869	0.004627	0.010712	0.005183	0.050361	0.013979
Verrucomicrobia	0.000067	0.000108	0.02175	0.000049	0.00013	0.008815
Proteobacteria	0.017271	0.008103	0.011887	0.021567	0.028466	0.010948
unidentified_Bacteria	0.003476	0.003045	0.003506	0.007415	0.004774	0.010682
Tenericutes	0.000189	0.000571	0.002671	0.003063	0.00009	0.000529
Deferribacteres	0.000191	0.001223	0.001285	0.000238	0	0.000064
Melainabacteria	0.000226	0.000313	0.000714	0.000045	0.000027	0.001674
Cyanobacteria	0.000019	0.000024	0.000032	0	0.000211	0.000121
Others	0.000294	0.000172	0.000587	0.000067	0.000103	0.00003

Table S2 Relative abundance of microbial species of the top 10 Genus in the feces of mice

	NCD	HFD	ITF	NCDG	HFDG	ITFG
Lactobacillus	0.26391	0.357975	0.132501	0.040085	0.102223	0.115728
Dubosiella	0.008313	0.096353	0.020311	0.016137	0.346637	0.028258
Alloprevotella	0.017532	0.012294	0.027851	0.081414	0.010788	0.002045
Bacteroides	0.013703	0.023213	0.036657	0.013928	0.002277	0.070631
unidentified_Clostridiales	0.000841	0.039549	0.001382	0.000481	0.001433	0.002223
Romboutsia	0.018368	0.004958	0.000132	0.001231	0.001828	0.000138
Akkermansia	0.000067	0.000108	0.02175	0.000049	0.00013	0.008815
Turicibacter	0.009445	0.029195	0.012135	0.016995	0.003993	0.004517
unidentified_Lachnospiraceae	0.007252	0.011682	0.019567	0.00918	0.007092	0.014815

Bifidobacterium	0.015635	0.000725	0.004239	0.002609	0.008313	0.011625
Others	0.644933	0.423949	0.723476	0.81789	0.515286	0.741205

Table S3 Concentrations of SCFAs in fecal samples of mice (x ± s, μmol/g)

**SCFAs**	**Group**	**Time**	
		**Before mating**	**GD18**
Acetic acid	NCD	5.32±0.93	5.05±1.60
	HFD	3.86±0.57	2.97±0.76
	ITF	5.60±1.12*	8.63±1.82**
Propionic acid	NCD	1.78±0.19	1.69±0.43
	HFD	1.67±0.18	1.73±0.60
	ITF	1.66±0.07	2.03±0.29
Butyric acid	NCD	2.15±0.23	2.03±0.28
	HFD	1.97±0.42	1.57±0.15
	ITF	2.05±0.30	3.32±0.61*

*P <0.05 vs HFD group; **P <0.01 vs HFD group
